# A new transmission test for affected sib-pair families

**DOI:** 10.1186/1753-6561-1-s1-s32

**Published:** 2007-12-18

**Authors:** Hongyan Xu, Varghese George

**Affiliations:** 1 Department of Biostatistics, Medical College of Georgia, 1120 15^th ^Street, AE-3031, Augusta, Georgia 30912, USA

## Abstract

Family-based association approaches such as the transmission-disequilibrium test (TDT) are used extensively in the study of genetic traits because they are generally robust to the presence of population structure. However, these approaches necessarily involve recruitment of families, which is more costly and time-consuming than sampling unrelated individuals in the population-based approaches. Therefore, a family-based approach, which has high power, would be appealing because of the gain in time and cost due to the reduced sample size that is required to attain adequate power. Here we introduce a new family-based transmission test using the joint transmission status from affected sib pairs. We show that by including the transmission status of both siblings, our method gives higher power than the TDT design, while maintaining the correct type I error rate. We use the simulated data from affected sib-pair families with rheumatoid arthritis provided by Genetic Analysis Workshop 15 to illustrate our approach.

## Background

Genetic association studies have contributed significantly in recent years to our understanding of the genetic basis of complex diseases. Association studies are roughly categorized into either population-based or family-based association approaches. Population-based approaches have the advantage that samples are easy to ascertain. However, it has been shown that population-based approaches, such as case-control studies, can produce spurious associations in the presence of population substructure, especially in large-scale studies at the genomic level [1,2]. In the presence of population substructure, family-based approaches, such as the transmission-disequilibrium test (TDT) originally proposed by Spielman et al. [3], have the advantage that they are robust against population substructure. Over the past decade or so, the original TDT has been extended and expanded to cover many practical scenarios, as alternative approaches to population-based association studies. Some of these extensions include the sibling-TDT [4], the homozygote parent-TDT [5], the pedigree disequilibrium test (PDT) [6,7], the quantitative TDT [8,9], the Bayesian TDT [10], an entropy-based method [11], and the more general family-based association test (FBAT) [12]. The motivation for these alternatives is that they are robust against population substructure and other cryptic relatedness in the samples [3,13]. However, these methods necessarily involve the recruitment of families, which may be more costly and time-consuming than sampling of unrelated individuals in population-based approaches. Therefore, a family-based approach, which has high power (consequently requiring a smaller sample size to achieve the same power) will be preferable. In this study we introduce a new family-based transmission test that is more powerful than the standard TDT, incorporating pair-wise transmission status of siblings. We demonstrate our approach using the simulated data from affected sib-pair (ASP) families with rheumatoid arthritis (RA) provided by Genetic Analysis Workshop 15 (GAW15). The simulated ASP families contain the genotypes of the parents, homozygous by heterozygous for the marker locus, and two affected siblings, from which the transmission status of both siblings from the heterozygous parent can be inferred. We show that by considering the transmission status of both siblings, our method gives the correct type I error rate while yielding higher power than the standard TDT design.

## Methods

Each of the 1500 simulated ASP families consists of four members: the father, the mother, and the ASP. The genotypes of all four members in each family are available. We used the simulated genotype data at both the genome-wide single-nucleotide polymorphism (SNP) markers and the chromosome 6 dense SNP markers from simulation Replicate 1.

**Figure 1:**
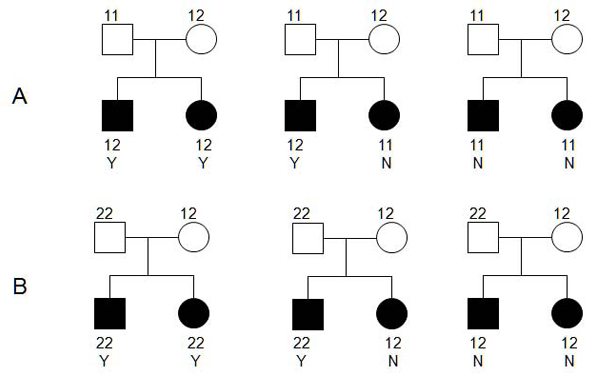
**Illustration of transmission status for ASP families**. Here we consider the transmission status of allele 2. Panel A is the possible transmission statuses of families with 11 by 12 mating; Panel B is the transmission statuses of families with 22 by 12 mating.

All the SNP markers are biallelic, denoted as 1 or 2. Suppose allele 2 is associated with rheumatoid arthritis affection status. We used all the families with a homozygous by heterozygous mating at the marker locus. For illustration, consider the transmission status of allele 2 from a pair of homozygous by heterozygous parents. We could classify the ASPs into one of three categories, (Y, Y), (Y, N), and (N, N), where Y means that allele 2 is transmitted from the heterozygous parent to the offspring, and N means it is not transmitted. Under the null hypothesis of no association between allele 2 at the marker locus and the rheumatoid arthritis affection status, the transmission probability of allele 1 and 2 should be equal. Figure 1 illustrates informative transmissions in the ASP families. In the family on the left in panel A, the heterozygous parent transmits allele 2 independently to both Sib 1 and Sib 2. Assuming Mendelian transmission under the null hypothesis, each transmission has probability of 1/2, and therefore, the probability of transmission status (Y, Y) is 1/2* 1/2 = 1/4. Similarly, the probability of (N, N) equals 1/4, as in the family on the right in panel A. The middle family in panel A corresponds to transmission status of (Y, N), in which the heterozygous parent transmits allele 2 to only one of the two siblings. There are two ways in which this can happen, i.e., allele 2 is transmitted to Sib 1 but not to Sib 2, or allele 2 is transmitted to Sib 2 but not to Sib 1. Each of these cases has probability 1/4, and therefore, the probability of transmission status (Y, N) is 1/4 + 1/4 = 1/2. Thus, the probabilities of transmission status (Y, Y), (Y, N), and (N, N) for ASPs correspond to 1/4, 1/2 and 1/4, respectively, under the null hypothesis. The expected number of ASPs for each transmission status can be calculated based on the total number of informative families and the probability of transmission status of the ASPs under the null hypothesis. Consequently, for testing the association between the marker locus and affection status, we construct a chi-square test statistic, $\chi^{2}=\sum_{i=1}^{3}\frac{{\left(O_{i}-E_{i}\right)}^{2}}{E_{i}}$, where the summation is over the three transmission types and *O *and *E *represent the observed and expected frequencies of each type, respectively. The test statistic has a chi-square distribution with 2 degrees of freedom under the null hypothesis of no association.

## Results

We applied our method to both the simulated data set of 9187 genome-wide SNPs from Replicate 1 and the data set of 17,820 dense SNPs on chromosome 6. For comparison, we also applied the standard TDT to the same set of families with homozygote by heterozygote matings. In performing the TDT analysis, we treated the transmission/non-transmission of the alleles to the two siblings in an ASP as two independent observations. This assumption may not necessarily be valid, and could result in inflated power.

### Type I error rate

The type I error rate is estimated empirically by performing the association tests on markers that are not associated with RA status and calculating the proportion of times the null hypothesis is rejected. We excluded the markers on the chromosomes that have trait loci associated with RA status and combined the test results of all available markers from all other chromosomes. Specifically, trait loci DR, C, and D on chromosome 6, locus F on chromosome 11, and locus E on chromosome 18 are associated with RA status, and, therefore, we used markers from all chromosomes excluding 6, 11 and 18, resulting in 7718 SNP markers for type I error analysis. Out of the 7718 tests performed using our approach and the standard TDT, our test rejected the null hypothesis 328 times, and the standard TDT rejected the null hypothesis 367 times. The corresponding estimated type I error rates of our method and TDT are 0.043 and 0.048, respectively, both of which are well under the nominal level of 0.05.

### Power

**Figure 2:**
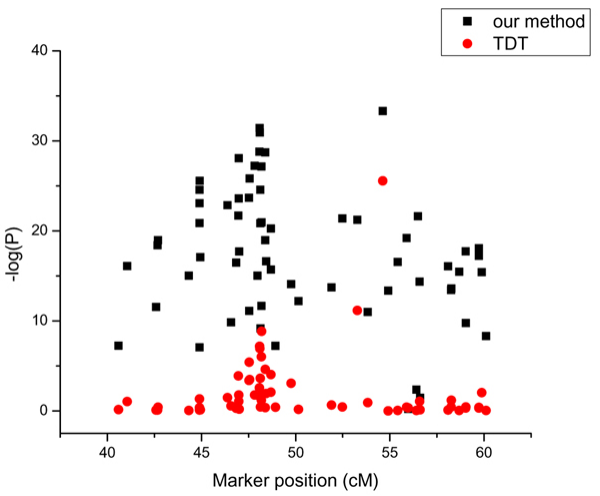
Plot of -log(P) against marker position around DR/C locus with genome-wide SNPs.

The power estimates of the two procedures are also obtained empirically by performing the test on markers that are known to be associated with the RA status. We used markers that are physically close to trait loci DR, C, and E on chromosome 6 for the power analysis. We performed the tests using our approach and the standard TDT on markers, and tabulated the number of times the null hypothesis of no association was rejected under various significance levels. These results are tabulated separately for the genome-wide SNPs and the dense SNPs on chromosome 6 in Tables 1 and 2. As is evident from these tables, our proposed method consistently detects true signals substantially more frequently than the standard TDT method. For example, in the analysis of genome-wide SNPs our approach detected 289 signals while the TDT detected 11, at the 0.0001 significance level. The corresponding numbers for the dense SNPs analysis are 7100 and 314, respectively. Both our method and the TDT could detect association signals with markers very close to trait loci DR, C, D, E, and F, even though the signals were consistently stronger using our approach (smaller *p*-values). However, as the distance between the trait loci and the marker increases, our approach consistently performs better than the TDT in detecting true signals. The pattern of test results on chromosome 6 for the two methods is given in Figure 2 in which -log(P) is plotted against the markers positions around DR/C loci. As Figure 2 suggests, our method gives smaller *p*-values, and correspondingly higher power, than the TDT throughout the entire region. Our method can also detect signals in an extended area, while the TDT detects signals only in the immediate vicinity of the trait loci. A similar pattern was also observed in the analysis of dense markers on chromosome 6. This suggests that in practice, if the makers are not very close the trait loci, we may fail to detect the signal using the TDT, while we can detect the signal with our method.

**Table 1:** Number of signals detected at various significance levels in the analysis of genome wide SNPs on chromosome 6

	Number of signals	
	
á	Our method	TDT
1.00×10^-10^	122	6
1.00×10^-5^	289	11
1.00×10^-3^	406	20
1.00×10^-2^	465	33

**Table 2:** Number of signals detected at various significance levels in the analysis of dense SNPs on chromosome 6

	Number of signals	
	
á	Our method	TDT
1.00×10^-10^	3307	150
1.00×10^-5^	7100	314
1.00×10^-3^	10147	527
1.00×10^-2^	12037	863

## Discussion

Because of the extra cost of recruiting family members, it is desirable to develop a family-based association method with relatively high power so that a smaller sample size is needed to achieve the same power. In this paper, we developed a new family-based transmission test using the joint transmission status of ASPs instead of the transmission status of individual offspring. The method gives the correct type I error rate and is more powerful than the TDT. In order to compare our method with the standard TDT, we treated the transmissions to the two siblings as two independent observations, which may not be a valid assumption for testing association [14]. The TDT is generally applicable to only parent-offspring trio data. Using more than one offspring may result in inflation of power because the sibship correlation is not taken into account, and the effective sample size is inflated. If we used only one offspring per ASP family for the TDT, then the number of positive signals detected using the standard TDT would have been even smaller because of the drop in sample size and resulting reduction in power. By considering the joint transmission statuses of two siblings, we, in fact, gained more power compared with the standard TDT, even in the case in which the two transmissions are erroneously treated as independent. The increased power is critical for the study of complex diseases, because it could reduce the necessary sample size, which is especially important for late-onset diseases in which recruiting families could be difficult. A disadvantage of our approach compared with the standard TDT is that it requires sib-pair data instead of singletons. However, the proposed method is a complementary approach to the traditional TDT when sib-pair family data are available. Further, it should be easy to combine the two approaches when both singletons and sib pairs are available.

It should be noted that the proposed approach, as well as the standard TDT, can easily be extended to include homozygous offspring from double heterozygous parents [10]. Also, both methods can be extended to include unaffected offspring and sib pairs, when available [10].

## Conclusion

We proposed a new family based transmission test using the simulated ASP family data from GAW15. Our method gives the correct type I error rate. By considering the transmission status of the two siblings simultaneously, our method has higher power than the standard TDT.

## Competing interests

The author(s) declare that they have no competing interests.
